# Rnd3 suppresses endothelial cell pyroptosis in atherosclerosis through regulation of ubiquitination of TRAF6

**DOI:** 10.1002/ctm2.1406

**Published:** 2023-09-24

**Authors:** Yan Zhang, Zhengru Zhu, Yang Cao, Zhenyu Xiong, Yu Duan, Jie Lin, Xuebin Zhang, Mengyuan Jiang, Yue Liu, Wanrong Man, Tengfei Jia, Jiaxu Feng, Yanyan Chen, Congye Li, Baolin Guo, Dongdong Sun

**Affiliations:** ^1^ Department of Cardiology Xijing Hospital, Fourth Military Medical University Xi'an China; ^2^ Department of Otolaryngology Xijing Hospital, Fourth Military Medical University Xi'an China

**Keywords:** atherosclerosis, endothelial cell, pyroptosis, Rho‐GTPase, ubiquitination

## Abstract

**Background:**

As the main pathological basis for various cardiovascular and cerebrovascular diseases, atherosclerosis has become one of the leading causes of death and disability worldwide. Emerging evidence has suggested that Rho GTPase Rnd3 plays an indisputable role in cardiovascular diseases, although its function in atherosclerosis remains unclear. Here, we found a significant correlation between Rnd3 and pyroptosis of aortic endothelial cells (ECs).

**Methods:**

Apoe^KO^ mice were utilized as a model for atherosclerosis. Endothelium‐specific transgenic mice were employed to disrupt the expression level of Rnd3 in vivo. Mechanistic investigation of the impact of Rnd3 on endothelial cell pyroptosis was carried out using liquid chromatography tandem mass spectrometry (LC‐MS/MS), co‐immunoprecipitation (Co‐IP) assays, and molecular docking.

**Results:**

Evidence from gain‐of‐function and loss‐of‐function studies denoted a protective role for Rnd3 against ECs pyroptosis. Downregulation of Rnd3 sensitized ECs to pyroptosis under oxidized low density lipoprotein (oxLDL) challenge and exacerbated atherosclerosis, while overexpression of Rnd3 effectively prevented these effects. LC‐MS/MS, Co‐IP assay, and molecular docking revealed that Rnd3 negatively regulated pyroptosis signaling by direct interaction with the ring finger domain of tumor necrosis factor receptor‐associated factor 6 (TRAF6). This leads to the suppression of K63‐linked TRAF6 ubiquitination and the promotion of K48‐linked TRAF6 ubiquitination, inhibiting the activation of NF‐κB and promoting the degradation of TRAF6. Moreover, TRAF6 knockdown countered Rnd3 knockout‐evoked exacerbation of EC pyroptosis in vivo and vitro.

**Conclusions:**

These findings establish a critical functional connection between Rnd3 and the TRAF6/NF‐κB/NLRP3 signaling pathway in ECs, indicating the essential role of Rnd3 in preventing pyroptosis of ECs.

## Graphical abstract

Rnd3 suppresses endothelial cell pyroptosis through regulation of ubiquitination of TRAF6. Rnd3 leads to the suppression of K63‐linked TRAF6 ubiquitination and the promotion of K48‐linked TRAF6 ubiquitination, inhibiting the activation of NF‐κB and promoting the degradation of TRAF6. Rnd3 interacted with the ring finger domain of TRAF6. The Rnd3/TRAF6/NF‐κB/NLRP3 signaling pathways in endothelial cell pyroptosis are triggered by oxLDL.

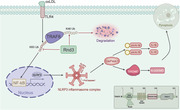

## 1 INTRODUCTION

Arteriosclerosis represents a chronic inflammatory process with abnormal proinflammatory response in the vasculature as a cardinal manifestation, in conjunction with lipid deposition, plaque formation, and intimal thickening as other primary confounding pathologies.^1^ As the main pathological basis for cardiovascular and cerebrovascular diseases, atherosclerosis has emerged as a prominent contributor to mortality and disability across the globe.^2^ Among all stakeholders for the etiology of atherosclerosis, endothelial cells (ECs) constitute not only a physiological barrier between peripheral tissues and the circulatory system, but also circulating signal transducers influencing vascular tone and permeability, oxidative stress, and platelet and leukocyte function, ultimately governing homeostasis in the vasculature.^3^ Growing evidence suggests that EC injury serves as the initial and vital event of atherosclerosis.^4^ Various pathophysiological stimuli can activate ECs, leading to the production of chemokines, cytokines, and adhesion molecules that attract inflammatory cells. In consequence, synthesis and secretion of nitric oxide (NO) from activated endothelial cells are reduced, leading to contractile and diastolic dysfunction of blood vessels. Meanwhile, chronic pro‐inflammatory state increases endothelial cell permeability, resulting in accumulation of leukocytes and lipids in the arterial intima, and eventually formation of foam cells and fatty streaks.^5^

Pyroptosis is a new form of programmed necrosis, in which the morphological changes are characterized by cell swelling with bubble‐like protrusions until the ultimate rupture of cell membrane, resulting in the release of cellular contents to trigger a profound proinflammatory response.^6^ The inflammasome sensors receive pathological signals and activate caspase‐1 through the NLR family pyrin domain containing 3 (NLRP3) inflammasome.^7^ Caspase‐1 cleaves gasdermin‐D (GSDMD) into the C‐terminal repressor domain and N‐terminal (GSDMD‐N) pore‐forming domain, which combines with the phospholipid protein on cell membrane, forms pore, and promotes release of cell contents to evoke pyroptosis. Caspase‐1 also cleaves the precursor molecules of IL‐1β and IL‐18 to generate active forms of these cytokines, which are then released into the extracellular space and can elicit proinflammatory responses.^8^

Rnd3, also known as RhoE, is a small GTP‐binding protein that belongs to the Rho family.^9^ Despite having GTPase structure and being able to bind the nucleotide, it lacks GTPase activity and therefore cannot hydrolyze GTP‐bound nucleotide. Earlier studies have shown that Rnd3 mainly acts as a suppressor for RhoA/ROCK1 signaling and participates in the regulation of cytoskeletal proteins.^9^ Recent studies have revealed several novel functions of Rnd3 in the governance of oxidative stress, inflammation, and lipid metabolism, indicating a diverse regulatory property for Rnd3 independent of RhoA/ROCK1.^10^ Rnd3 has shown overt protective effects in a wide range of cardiovascular diseases, including myocardial infarction, hypertension, arrhythmias, and cardiomyopathy, denoting its translational potential as an intervention target.^11^, ^12^, ^13^, ^14^ As for ECs, Rnd3 has been identified as a hub gene that causes peripheral venous congestion and EC injury.^15^ Recent research has indicated that Rnd3 has potential properties to promote the restoration of endothelial barriers and act as an anti‐inflammatory agent by rebalancing RhoA and Rac1 signaling.^16^ Furthermore, Rnd3 has been found to suppress inflammation after myocardial infarction,^11^ offering a promising strategy for mitigating chronic inflammation in the progression of atherosclerosis. However, the role of Rnd3 in atherosclerosis has not been adequately investigated, making it essential to establish a theoretical foundation for its involvement.

Tumor necrosis factor receptor‐associated factor 6 (TRAF6) is a signaling molecule common to both the IL‐1R/TLR family and the TNFR superfamily, which is linked with tumor necrosis factor (TNF) receptors.^17^ TRAF6 is composed of a RING finger (RF) domain sequence at the N‐terminal and five continuous zinc finger (ZF) sequences in the middle, followed by a coiled helix and a conserved C‐terminal domain of TRAFs.^18^ TRAF6 mediates a wide range of protein–protein interactions through its RF domain containing nonconventional E3 ubiquitin ligase function.^19^ K48‐ and K63‐linked ubiquitin chains are the two most abundant forms of ubiquitin chains.^17^, ^20^ TRAF6 can bind with TGFβ‐activated kinase 1 (TAK1) and Tak1‐binding proteins via k63 ubiquitin chains and activates the inhibitory kappa B kinase (IKK), which in turn promotes phosphorylation of inhibitor of nuclear factor κBα (IκBα).^21^ In addition, TRAF6 undergoes degradation via the 26S proteasome as a result of K48‐linked ubiquitination.^22^

Here, we noted a previously unrecognized Rnd3‐dependent signaling pathway regulating endothelial pyroptosis via its direct interaction with TRAF6. Rnd3 induces degradation of TRAF6 through K48‐linked ubiquitination to prevent TAK1 complex formation and subsequent activation of NF‐κB pathway through K63‐linked ubiquitination. These results should help to establish a novel functional link between Rnd3 and TRAF6/NF‐κB/NLRP3 signaling axis in ECs, thus favoring a fundamental role for Rnd3 in the combat of pyroptosis of ECs.

## 2 METHODS

### 2.1 Cell culture and treatment

Mouse aortic ECs were isolated as described previously with modifications.^38^, ^39^ After anesthesia, aorta was isolated from mice under aseptic conditions and were placed in 10% PBS with penicillin‐streptomycin solution. Following removal of fat and connecting tissues, the lumen of aorta was flushed twice using Phosphate buffered saline (PBS) and was placed in endothelial culture medium (ScienCell) containing 10% Fetal bovine serum (FBS). Aorta were ligated at the tip with surgical sutures and the bottom end was connected by a hose onto a 5‐mL syringe containing 3 mL of type II collagenase (2 mg/mL; Invitrogen), which was injected at a rate of 150 μL per minute by a syringe pump (LSP04‐1A, Longer Precision Pump Co., Ltd.). After 20 min, the medium in the lumen was collected, centrifuged at 1000 rpm for 5 min, and resuspended by the same medium. Endothelial cells isolated from every three aortas were pooled and seeded in a six‐well plate. Cellular purity was verified using immunofluorescence staining of CD31. Cells were incubated at 37°C, 5% CO_2_, and passaged until they reached 80%–90% confluence. Only endothelial cells after two to three passages were used in this study. The pyroptosis of ECs were induced by treatment with 25 μg/mL oxLDL (Yiyuan Biotechnologies) for 24 h.

Peritoneal macrophages were collected as previously described. Briefly, male mice aged 6–8 weeks were intraperitoneally delivered 1 mL sterile 3% (wt/vol) thioglycollate broth. After 72 h, 5 mL sterile PBS was intraperitoneally injected and samples were collected 5 min later. The peritoneal cavity was rinsed with the same volume of PBS, with perfusate collected. The peritoneal macrophages were collected by centrifugation at 1000 rpm for 5 min and were suspended in fresh RPMI 1640 medium containing 10% FBS prior to culture for further experimentations.

### 2.2 Oil Red O staining

Following euthanasia, aorta and heart were separated prior to fixation in 4% paraformaldehyde for 24 h. The aorta was cut lengthwise prior to placement in an Oil Red dye solution at 37°C for 60 min. Then fatty plaques were differentiated with 75% ethanol until they turned bright red and aorta almost colorless. Aorta was rinsed twice with distilled water. Heart tissues dehydrated with 30% sucrose were embedded in the Optimal cutting temperature (OCT) compound and the section was collected at the root of aorta (8‐μm thickness). Sections were stained with Oil Red O (Sigma‐Aldrich) for lipids as described.^40^

### 2.3 Flow cytometry

For pyroptosis assay, ECs with various treatments were evaluated using flow cytometry (COULTER EPICS XL‐MCL; Beckman Coulter). The Caspase‐1 Assay Kit (ImmunoChemistry Technologies) was utilized to detect the active Caspase‐1 enzyme within live cells. Propidium iodide (Beyotime Biotechnology) was employed to identify the dead cells in a given cell population. Unstained cells were used as control to determine specific Forward scatter/Side scatter (FSC/SSC) gates used in this test.

### 2.4 Evaluation of related protein expression

Western blot and immunofluorescence staining were utilized to assess associated proteins, following the same experimental procedures previously described.^41^, ^42^ The detailed operation procedures and related antibodies (Table S1) are provided in the Supporting Information.

### 2.5 Co‐immunoprecipitation

Co‐IP was carried out utilizing an IP/Co‐IP Kit (Thermo Fisher Scientific) as per the manufacturer's instructions. Initially, cellular lysates comprising 500 μg of protein were mixed with 5 μg of IP antibody per sample in a microcentrifuge tube and incubated for 12 h at 4°C. Subsequently, the antigen/antibody complex was allowed to attach to Protein A/G magnetic beads for 1 h at 37°C. The beads were washed twice with an IP Lysis/Wash Buffer and once with purified water. The protein was collected and used for western blotting, and the Clean‐Blot IP (Thermo Fisher Scientific) was utilized to prevent interference from the heavy chain and light chain segments of the antibody following the protocol.

### 2.6 Silver staining

To discern Rnd3‐driven protein interaction, proteins interacting with Rnd3 were electrophoretic separated and visualized using a Fast Silver Stain Kit (Beyotime Biotechnology) per manufacturer's instructions. Briefly, the gel was fixed with fixative to remove interfering ions and detergent. Second, sensitizer was employed to enhance sensitivity and contrast of the staining dye. Finally, developer was used to convert the silver ions into metallic silver on the gel bands.

### 2.7 Assessment of macrophage migration and phagocytosis

Transwell chemotactic assay was employed to evaluate the migration of macrophage. The medium of ECs with various treatments was collected after 24 h. The ECs condition medium was added underneath the cell permeable membrane of transwell (Corning), and equal numbers of macrophages were cultivated on the upper layer of the transwell. The cells that had migrated across the membrane were stained with crystal violet after 48 h and the respective cell count was observed under a microscope.

Macrophage oxLDL engulf assay was used to evaluate the phagocytosis of macrophage. Macrophages were cultured with ECs condition medium from different groups for 24 h, then the medium was replaced with a complete medium contain oxLDL (50 μg/mL) for another 24 h. Finally, macrophages were fixed with 4% paraformaldehyde and were stained with Oil Red.

### 2.8 Molecular docking

The FAST sequence of mouse TRAF6 was downloaded from Uniprot (https://www.uniprot.org) and homologous modeling was performed by AlphaFold 2. The protein structure of Rnd3 (PDB ID: 1GWN) was downloaded from the PDB database (https://www.rcsb.org). The interaction mode between TRAF6 and Rnd3 was performed using Zdock (https://zdock.umassmed.edu). Pymol 2.3.0 was utilized to evaluate the interaction mode of the docking results.

### 2.9 Gene intervention

At the in vivo level, we utilized transgenic mice and adeno‐associated viruses to interfere with the expression of genes that were relevant to our study. At the in vitro level, we used adenovirus to disturb the expression of genes that were related to our research. The Supporting Information provides details on the construction process and feeding method of the transgenic mice, the injection method of the adeno‐associated virus at the in vivo level, and the transfection method of the adenovirus at the in vitro level.

### 2.10 Statistical analysis

Data are expressed as mean ± standard deviation of the mean (SD). The Shapiro–Wilk test was used to determine data normality. Unpaired Student's *t*‐test was employed to assess difference between two groups. Differences between multiple groups were evaluated using one‐way analysis of variance followed by the Tukey's post hoc test. All statistical analyses were performed using GraphPad Prism 8 (GraphPad Software, CA). A *p* value of <.05 was deemed statistically significant.

## 3 RESULTS

1. Apoe^KO^ mice present evident pyroptosis and downregulated Rnd3 levels in endothelium of atherosclerotic lesion

**FIGURE 1:**
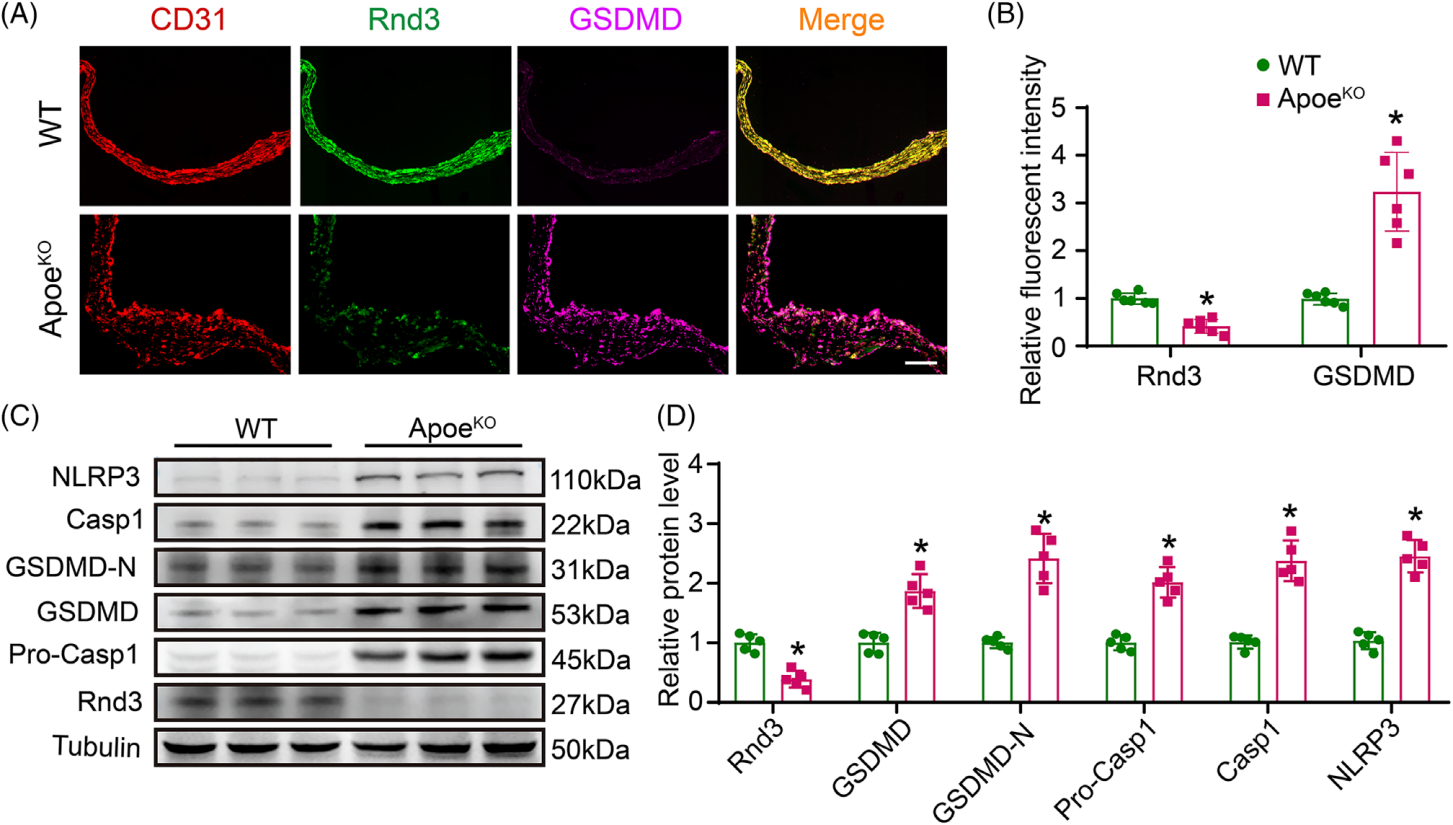
Apoe^KO^ mice present increased pyroptosis and reduced Rnd3 levels in endothelial cells (ECs) of atherosclerotic lesion. (A) The protein level of Rnd3 and gasdermin‐D (GSDMD) was evaluated using immunofluorescence staining in the aortic roots of wild‐type (WT) mice and Apoe^KO^ mice (*n* = 6). Scale bars represent 200 μm. (B) Quantification of the fluorescent intensity of Rnd3 and GSDMD immunofluorescence staining in ECs of wild‐type (WT) mice and Apoe^KO^ mice. (C) The expression of Rnd3, NLRP3, GSDMD, GSDMD‐N, Caspase1 and Pro‐Caspase 1 in different groups by western blot (*n* = 5). (D) Quantitative analysis of Rnd3, NLRP3, GSDMD, GSDMD‐N, Caspase 1, and Pro‐Caspase1 protein levels in different groups. *p < .05 versus WT.

To evaluate whether endothelial Rnd3 is involved in the pathological progression of atherosclerosis, we performed immunofluorescence staining at the aortic roots of wild type (WT) and Apoe^KO^ mice, following high‐fat diet (HFD) feeding for 8 weeks. The expression of GSDMD was significantly increased in ECs of Apoe^KO^ mice, while Rnd3 was decreased in comparison with WT mice. CD31 was used to label ECs (Figure 1A,B). Furthermore, western blot data revealed that GSDMD‐N was also increased in aortic ECs isolated from Apoe^KO^ mice, compared with those from WT mice. Moreover, the decreased protein level of Rnd3 and increased NLRP3 and Caspase1 in Apoe^KO^ mice was demonstrated by western blot (Figure 1C,D). These observations favor a potential role of Rnd3 in atherosclerosis pathogenesis.

2. Overexpressing of Rnd3 inhibited endothelial pyroptosis and atherosclerosis in Apoe^KO^ mice

**FIGURE 2:**
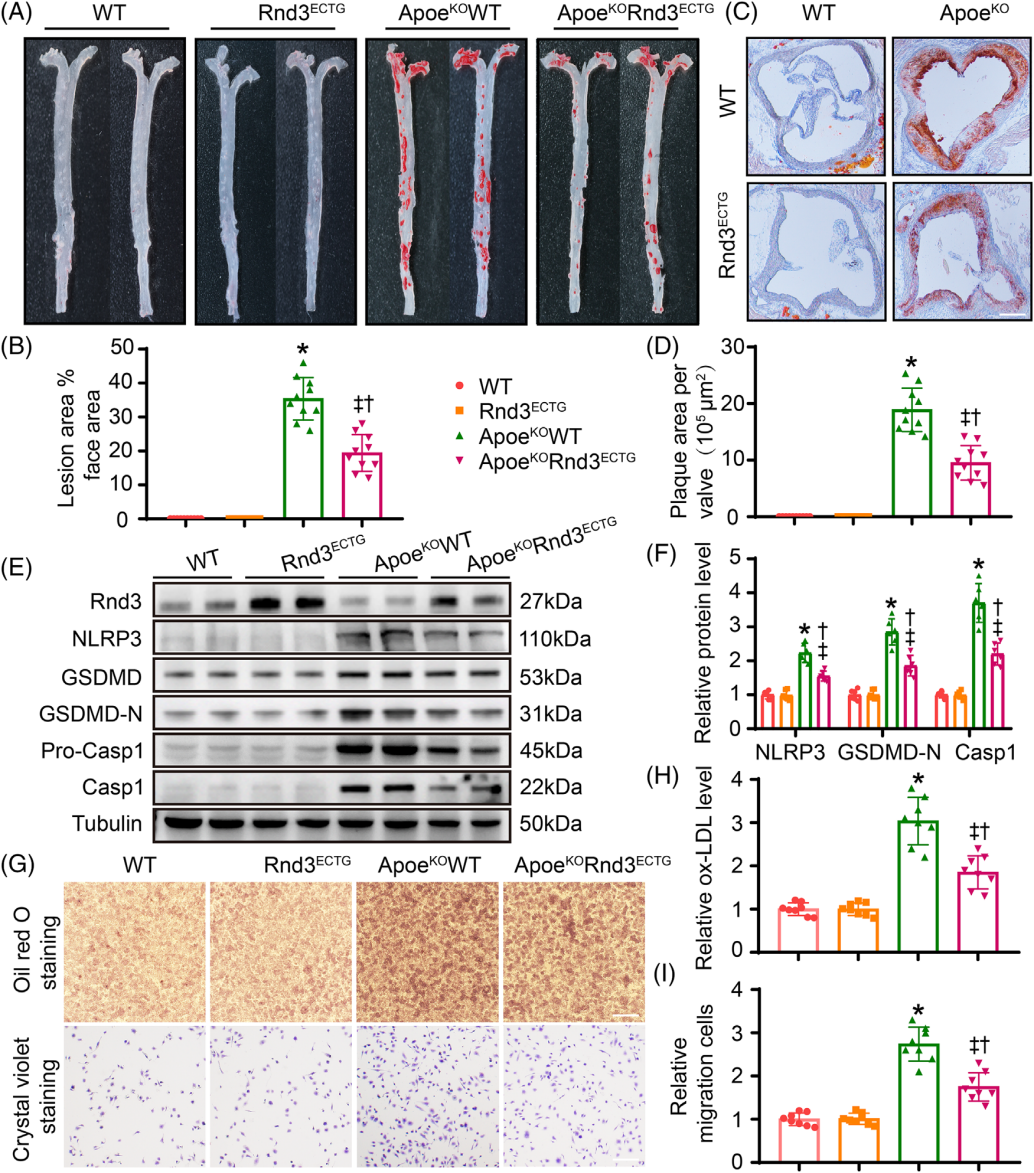
Overexpression of Rnd3 inhibits endothelial pyroptosis and atherosclerosis in Apoe^KO^ mice. (A) Representative Oil Red O staining of aorta in different groups (*n* = 10). (B) Quantification of the percentage of aortic lesion areas in different groups. (C) Representative Oil Red O staining of aortic roots in different groups (*n* = 10). Scale bars represent 500 μm. (D) Quantification of the percentage of lesion areas in aortic roots of different groups. (E and F) Representative and pooled western blot analysis of Rnd3 and pyroptosis‐associated proteins in different groups (*n* = 6). (G) Assessment of macrophage migration and phagocytosis by Oil Red O staining and crystal violet staining, which treated by condition medium of endothelial cells (ECs) from different groups (*n* = 8). Scale bars represent 200 μm. (H) Relative oxLDL levels in different groups were quantified. (I) Relative migration cells in different groups were quantified. *p < .05 versus WT; †*p* < .05 versus Rnd3ECTG; ‡*p* < .05 versus ApoEKOWT.

To determine the role of endothelial cell Rnd3 in atherosclerosis, Rnd3^ECTG^ mice were constructed and crossed with Apoe^KO^ mice to generate Apoe^KO^Rnd3^ECTG^ mice as described. PCR was used for mouse genotyping (Figure 1A). Western blot and immunofluorescence were employed to confirm the specificity of Rnd3 overexpression in ECs ( Figure 1B–D). Following 8 weeks of HFD treatment, Oil Red O staining revealed little plaque formation in WT and Rnd3^ECTG^ mice. Plaques were found in 36.3 ± 1.8% of total aorta of Apoe^KO^WT mice and 18.8 ± 1.4% of Apoe^KO^Rnd3^ECTG^ mice (*p* < .05) (Figure 2A,B). Similarly, overexpressing of Rnd3 in ECs decreased the plaque area of aortic roots in Apoe^KO^ mice (Apoe^KO^WT vs. Apoe^KO^Rnd3^ECTG^; 17.9 ± 1.3 10^5^ μm^2^ vs. 9.6 ± 1.2 10^5^ μm^2^, *p* < .05) (Figure 2C,D). As Rnd3 plays a crucial role in regulating adipocyte lipolysis, we first evaluated the triglyceride to high‐density lipoprotein cholesterol (TAG/HDL‐C) ratio in each group of mice. However, since our transgenic mice were endothelial specific, there was no significant difference in circulating TAG/HDL‐C levels between the groups (Figure S1E). On the other hand, Apoe^KO^Rnd3^ECTG^ mice displayed lower endothelial pyroptosis compared with Apoe^KO^WT mice, exemplified by lower expression levels of NLRP3, Pro‐caspase1, Caspase1, GSDMD, and GSDMD‐N in ECs (Figure 2E,F, Figure S1F–I).

It is well perceived that endothelial injury promotes monocyte recruitment and foam cell formation. Here, the impacts of EC‐Rnd3 on macrophage migration and phagocytosis were examined in vitro. As expected, peritoneal macrophages exhibited less oxLDL phagocytosis when treated by condition medium from ECs of Rnd3‐overexpressing aorta (Figure 2G,H). In addition, ECs prepared from aortas of Apoe^KO^Rnd3^ECTG^ mice displayed significantly decreased peritoneal macrophage migration in transwell (Figure 2I). Together, these results demonstrated a protective role for Rnd3 against atherosclerosis by inhibition of endothelial pyroptosis.

3. Rnd3 inhibited endothelial pyroptosis through regulation of TRAF6 ubiquitination

**FIGURE 3:**
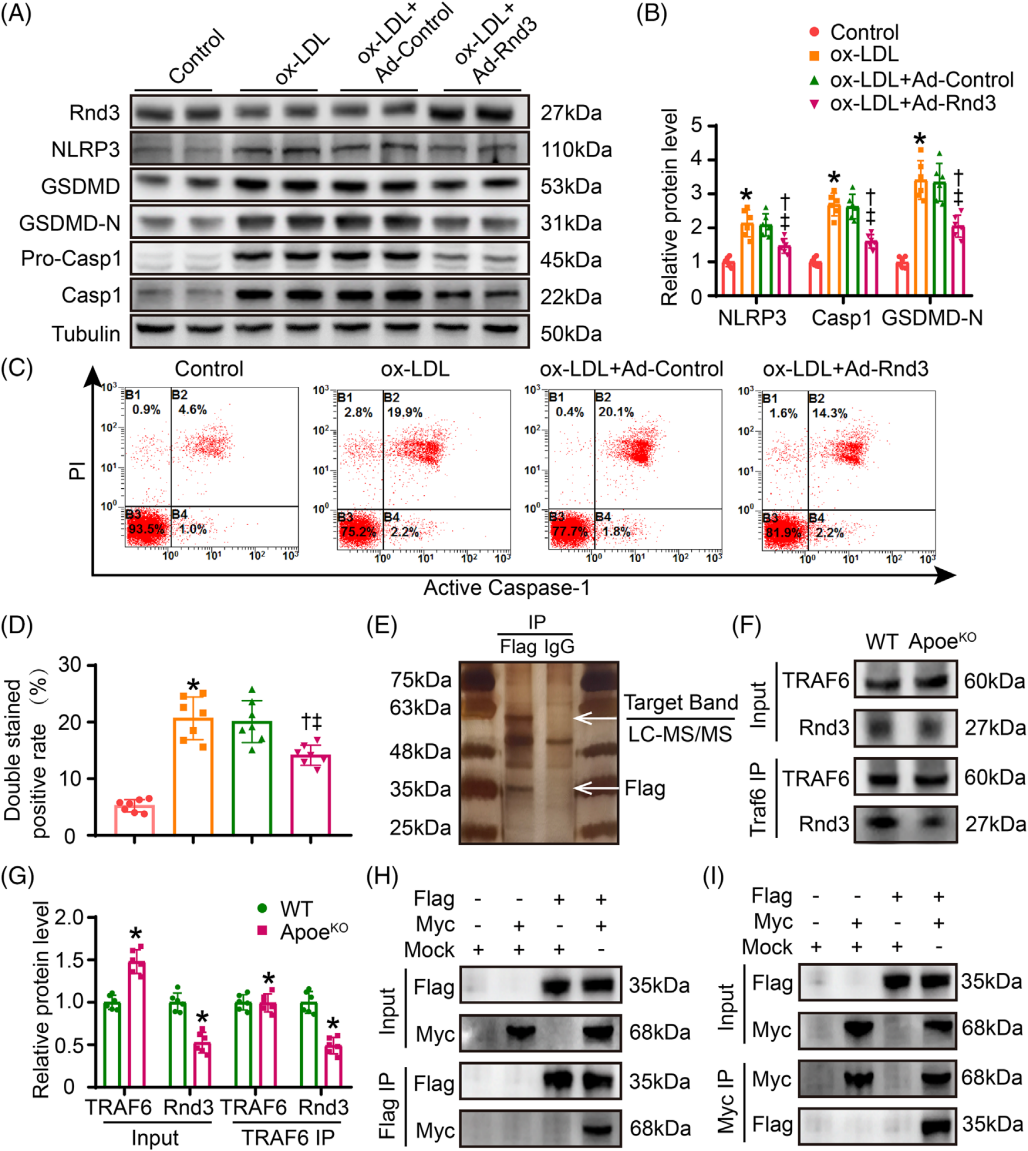
Rnd3 interacts with TRAF6 in endothelial cells (ECs). (A and B) Representative and pooled western blot analysis of Rnd3 and pyroptosis‐associated proteins in different groups (*n* = 6). (C and D) Flow cytometry and associated quantitative analysis of propidium iodide (PI) and Caspase‐1 double‐staining positive cells treated as indicated (*n* = 5). *p < .05 versus control; **^†^** *p* < .05 versus oxLDL; **^‡^** *p* < .05 versus oxLDL + Ad‐Control. (E) Flag antibody or IgG (negative control antibody) was used for immunoprecipitation, electrophoresed and silver staining (*n* = 5). (F and G) Interaction between Rnd3 and TRAF6 was demonstrated by immunoprecipitation in ECs of wild‐type (WT) mice and Apoe^KO^ mice (*n* = 6). *p < .05 versus WT. (H and I) Primary aortic ECs were transfected with mock, Ad‐Flag‐Rnd3, or Ad‐Myc‐TRAF6 as indicated and performed immunoprecipitation using anti‐Flag or anti‐Myc antibody.

To evaluate the role of Rnd3 in endothelial cells, primary aortic ECs were isolated from WT mice, and oxLDL was used to evoke endothelial pyroptosis in vitro. Ad‐Flag‐Rnd3 was transfected to ECs overexpressing Rnd3, and the adenoviral transfection efficiency was evaluated by western blot analysis (Figure S2A). As shown in Figure 3A, oxLDL significantly induced pyroptosis of ECs and displayed higher expression levels of NLRP3, Caspase1, and GSDMD‐N. However, Ad‐Flag‐Rnd3 treatment notably suppressed pyroptosis level of ECs under oxLDL challenge, in comparison with Ad‐control (Figure 3 A,B, Figure S2B–E). Flow cytometry further demonstrated that Rnd3 overexpression decreased the number of propidium iodide (PI) and Caspase‐1 double‐staining positive cells treated with oxLDL (Figure 3C,D).

To further explore Rnd3‐driven mechanisms in atherosclerosis, immunoprecipitation was performed in ECs treated with Ad‐Flag‐Rnd3 in the face of oxLDL challenge. The silver staining results showed a pronounced Flag‐Rnd3 band around 35 kDa. A distinct band was also detected below 63 kDa, which may contain key proteins interacting with Rnd3 (Figure 3E). After performing LC‐MS/MS analysis on the band below 63 kDa (Table S2), the protein TRAF6 was chosen for further investigation.

To verify potential interaction between Rnd3 and TRAF6 in ECs, immunoprecipitation was conducted in ECs isolated from WT and Apoe^KO^ mice. Consistent with our earlier results, the protein level of Rnd3 was downregulated in Apoe^KO^ mice, while the protein level of TRAF6 was increased in Apoe^KO^ mice. Following immunoprecipitation (IP) using TRAF6 antibody, a notable interaction was detected between Rnd3 and TRAF6, and the interaction weakened in Apoe^KO^ mice (Figure 3F,G). Second, primary aortic ECs were transfected with either a mock, Ad‐Flag‐Rnd3, or Ad‐Myc‐TRAF6 as directed. Note that 36 h post‐transfection, the cell lysates underwent co‐immunoprecipitation (Co‐IP) using either an anti‐Flag or anti‐MYC antibody, followed by immunoblotting using either an anti‐Flag or anti‐MYC antibody as well. The immunoprecipitation assay revealed that Flag‐Rnd3 proteins were precipitated with Myc‐TRAF6 protein, but not the mock (Figure 3H,I). Meanwhile, immunofluorescence was conducted to demonstrate the co‐localization of Rnd3 and TRAF6 in primary aortic ECs (Figure S2F).

**FIGURE 4:**
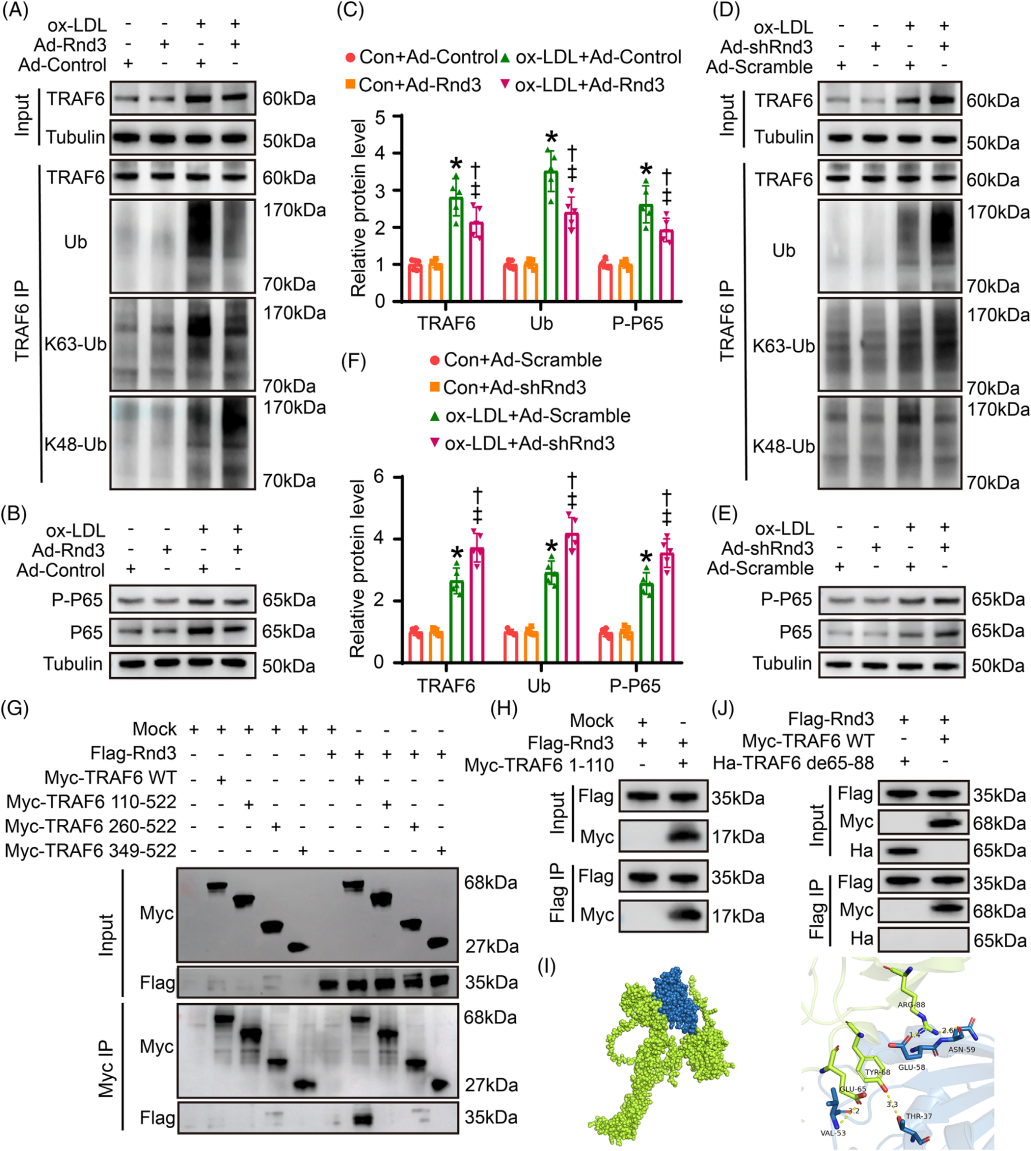
Rnd3 inhibits endothelial pyroptosis by regulating ubiquitination of TRAF6. (A) Effects of Rnd3 overexpression on TRAF6 ubiquitination. The protein level of TRAF6 in input, and Ub, K63‐Ub, and K48‐Ub after TRAF6 immunoprecipitation (IP) was evaluated by western blot (*n* = 5). (B) Effects of Rnd3 overexpression on NF‐κB activation. The protein level of P‐P65 and P65 was evaluated by western blot (*n* = 5). (C) Quantitative analysis of TRAF6, Ub, and P‐P65 protein levels in different groups. *p < .05 versus Con+Ad‐Control; **^†^** *p* < .05 versus Con+Ad‐Rnd3; **^‡^** *p* < .05 versus oxLDL+Ad‐Control. (D) Western blotting was used to evaluate the effect of Rnd3 knockdown on TRAF6 ubiquitination (*n* = 5). (E) Western blotting was used to evaluate the effect of Rnd3 knockdown on NF‐κB activation (*n* = 5). (F) Quantitative analysis of TRAF6, Ub, and P‐P65 protein levels in different groups. *p < .05 versus Con+Ad‐Scramble; **^†^** *p* < .05 versus Con+Ad‐shRnd3; **^‡^** *p* < .05 versus oxLDL+Ad‐Scramble. (G) Myc‐TRAF6 wild type (WT), Myc‐TRAF6 truncation mutants (110‐522, 260‐522, 349‐522), and Flag‐Rnd3 were transfected to endothelial cells (ECs) and performed immunoprecipitation using anti‐Myc antibody. (H) Myc‐TRAF6 truncation mutants (1‐110) and Flag‐Rnd3 were transfected to ECs and performed immunoprecipitation using anti‐Flag antibody. (I) Molecular docking of Rnd3 and TRAF6 combination. The potential interaction regions (TRAF6 65 to 88 amino acids) were found. (J) Flag‐Rnd3, Myc‐TRAF6 WT, or Ha‐TRAF6 65–88 amino acid deletion mutant was transfected to ECs and performed immunoprecipitation using anti‐Flag antibody.

TRAF6 plays a crucial role as a downstream regulator for several immunoregulatory receptors through its action as a ubiquitin ligase, capable of ubiquitinating with both K48‐linked degradative chains and K63‐linked non‐degradative chains at various sites.^17^ Whether Rnd3 regulates ubiquitination of TRAF6 remains unclear. Our results showed that EC Rnd3 overexpression significantly downregulated TRAF6 protein level under oxLDL challenge. Additionally, Rnd3 overexpression decreased total ubiquitination and K63 ubiquitination of TRAF6 while enhancing K48 ubiquitination (Figure 4A, Figure S2G,H). TRAF6 is targeted for degradation by proteasome with the covalent attachment of K48 ubiquitin chains.^23^ K63 ubiquitination is important for NF‐κB activation by activating the downstream TAK1.^24^ Mechanistically, decreased K63 ubiquitination caused by Rnd3 overexpression suppressed NF‐κB activation (Figure S4B,C). Conversely, Rnd3 knockdown enhanced K63 ubiquitination of TRAF6 and the activation of NF‐κB, attenuated K48 ubiquitination and expression level of TRAF6 (Figure 4D–F, Figure S2I–K). Moreover, the mutants of K48 and K63 resulted in a significant decrease in K48 and K63‐linked ubiquitination of TRAF6 under oxLDL challenge. This also effectively counteracted the regulatory influence of Rnd3 on both TRAF6 degradation and NF‐κB activation (Figure 3A–D). The ubiquitin‐proteasome system plays a crucial role in protein degradation. Inhibition of the proteasome using MG132 (MCE, 10 μM) effectively prevented the degradation of TRAF6 induced by Rnd3. However, inhibitors of autophagy and lysosomes (3‐MA, MCE, 5 mM; Lys05, MCE, 10 μM) showed no significant effect on the protein level of TRAF6 (s Figure 3E–G). These results suggest that Rnd3 regulates TRAF6 through both K48 and K63‐linked ubiquitination.

4. Rnd3 interacted with the RF domain of TRAF6

To further determine the specific mode of interaction between Rnd3 and TRAF6, Myc‐TRAF6 wild type (WT), Myc‐TRAF6 truncation mutants (110‐522, 260‐522, 349‐522), and Flag‐Rnd3 were transfected to ECs, and IP assay was performed using an anti‐Myc antibody. The result showed that Flag‐Rnd3 proteins were observably precipitated with Myc‐TRAF6 WT, but not with Myc‐TRAF6 truncation mutants, indicating that Rnd3 could interact with the RF domain of TRAF6 (1–110) (Figure 4G). To verify the aforementioned results, Flag‐Rnd3 and Myc‐TRAF6 (1‐110) were transfected into ECs, and an anti‐Flag antibody was used for IP assay. As expected, Flag‐Rnd3 proteins were markedly precipitated with Myc‐TRAF6 (1–110) (Figure 4H). Based on computer‐aided molecular docking, the potential interaction regions (TRAF6 65–88 amino acids) were found (Figure 4I). The truncation mutants based on this structure were constructed to identify key domains of Rnd3–TRAF6 interactions. IP assay further demonstrated that the truncation of TRAF6 protein in the 65–88 amino acid domain nullified the binding between TRAF6 and Rnd3 (Figure 4J). This finding suggests that the 65–88 amino acid domain of TRAF6 may serve as a key structure to target Rnd3. Together, Rnd3 regulates ubiquitination of TRAF6 by binding to the 65–88 amino acid domain, en route to activation of NF‐κB and pyroptosis of ECs.

5. Knockout of Rnd3 exacerbated endothelial pyroptosis and atherosclerosis in Apoe^KO^ mice

**FIGURE 5:**
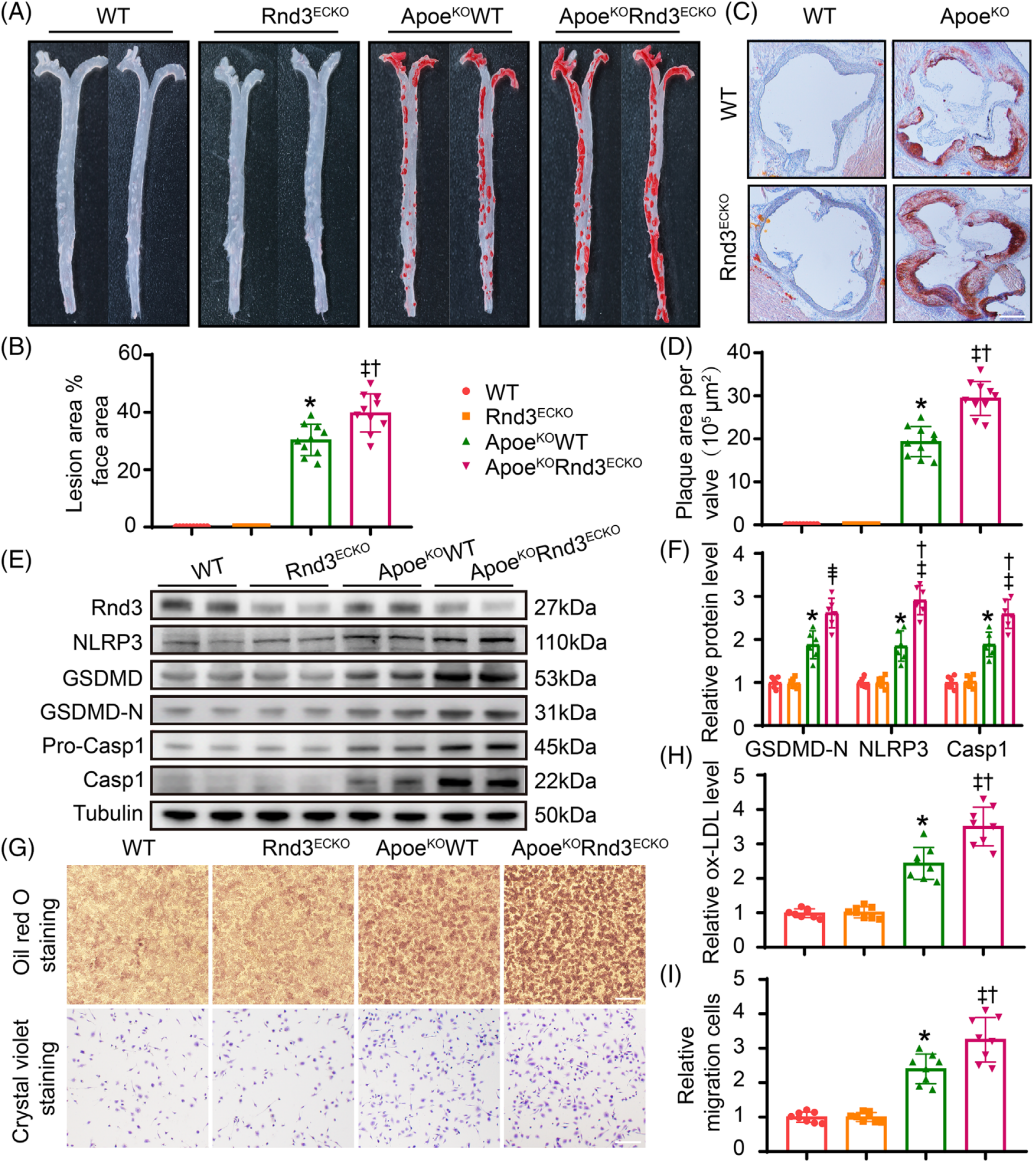
Knockout of Rnd3 exacerbates endothelial pyroptosis and atherosclerosis in Apoe^KO^ mice. (A) Representative Oil Red O staining of aorta in different groups (*n* = 10). (B) Quantification of the percentage of aortic lesion areas in different groups. (C) Representative Oil Red O staining of aortic roots in different groups (*n* = 10). Scale bars represent 500 μm. (D) Quantification of the percentage of lesion areas in aortic roots of different groups. (E and F) Representative and pooled western blot analysis of Rnd3 and pyroptosis‐associated proteins in different groups (*n* = 6). (G) Assessment of macrophage migration and phagocytosis by Oil Red O staining and crystal violet staining, which is treated by condition medium of ECs from different groups (*n* = 8). Scale bars represent 200 μm. (H) Relative oxLDL levels in different groups were quantified. (I) Relative migration cells in different groups were quantified. *p < .05 versus WT; †*p* < .05 versus Rnd3ECKO; **^‡^** *p* < .05 versus ApoE^KO^WT.

Having demonstrated the protective role of EC‐Rnd3 on atherosclerosis and the underlying mechanism, endothelial‐specific Rnd3 knockout mice (Rnd3^ECKO^) were generated and crossed with Apoe^KO^ mice to produce Apoe^KO^Rnd3^ECKO^ mice. PCR was employed to determine genotypes of experimental mice (Figure S4A). Following 8 weeks of HFD feeding, Oil Red O staining exhibited little plaque formation in WT and Rnd3^ECKO^ mice; plaques were found in 31.3 ± 1.8% of total aorta of Apoe^KO^WT mice and 39.4 ± 2.3% of total aorta of Apoe^KO^Rnd3^ECKO^ mice (*p* < .05) (Figure 5A,B). EC‐Rnd3 knockout in Apoe^KO^ mice also resulted in elevated aortic root plaque area, in comparison with Apoe^KO^ mice (Apoe^KO^WT vs. Apoe^KO^Rnd3^ECKO^; 18.6 ± 1.2 × 10^5^ μm2 vs. 28.8 ± 1.9 105 μm^2^, *p* < .05) (Figure 5C,D). To decipher pyroptosis in ECs, Rnd3 knockout in Apoe^KO^ mice significantly increased the expression levels of NLRP3, Caspase1, and GSDMD‐N (Figure 5E,F, Figure S4B–E). Furthermore, Rnd3 knockout in ECs facilitated macrophage migration and phagocytosis (Figure 5G–I). These results favor a key role for decreased Rnd3 levels in endothelial cells in the pathological progression of atherosclerosis.

6. TRAF6 knockdown offsets endothelial pyroptosis and atherosclerosis exacerbated by Rnd3 knockout

Finally, mouse TRAF6 knockdown adeno‐associated virus‐9 genome particles containing the TIE promoter, Flag, and enhanced green fluorescent protein (AAV9‐m‐TIE‐shTRAF6‐Flag‐EGFP, abbreviated as AAV9‐shTRAF6) were used to further confirm the regulatory role of Rnd3 on TRAF6. AAV9‐shTRAF6 or AAV9‐Scramble was injected into Apoe^KO^WT and Apoe^KO^Rnd3^ECKO^ mice through caudal veins at 8 weeks of age. After 4 weeks, double staining of Flag and CD31 indicated transfection efficiency of AAV9 in aortic endothelial cells >95% (s Figure 4F). Meanwhile, the TRAF6 protein level of AAV9‐shTRAF6‐transfected mouse ECs was 3.6 times lower than that of control group (Figure S4G,H).

**FIGURE 6:**
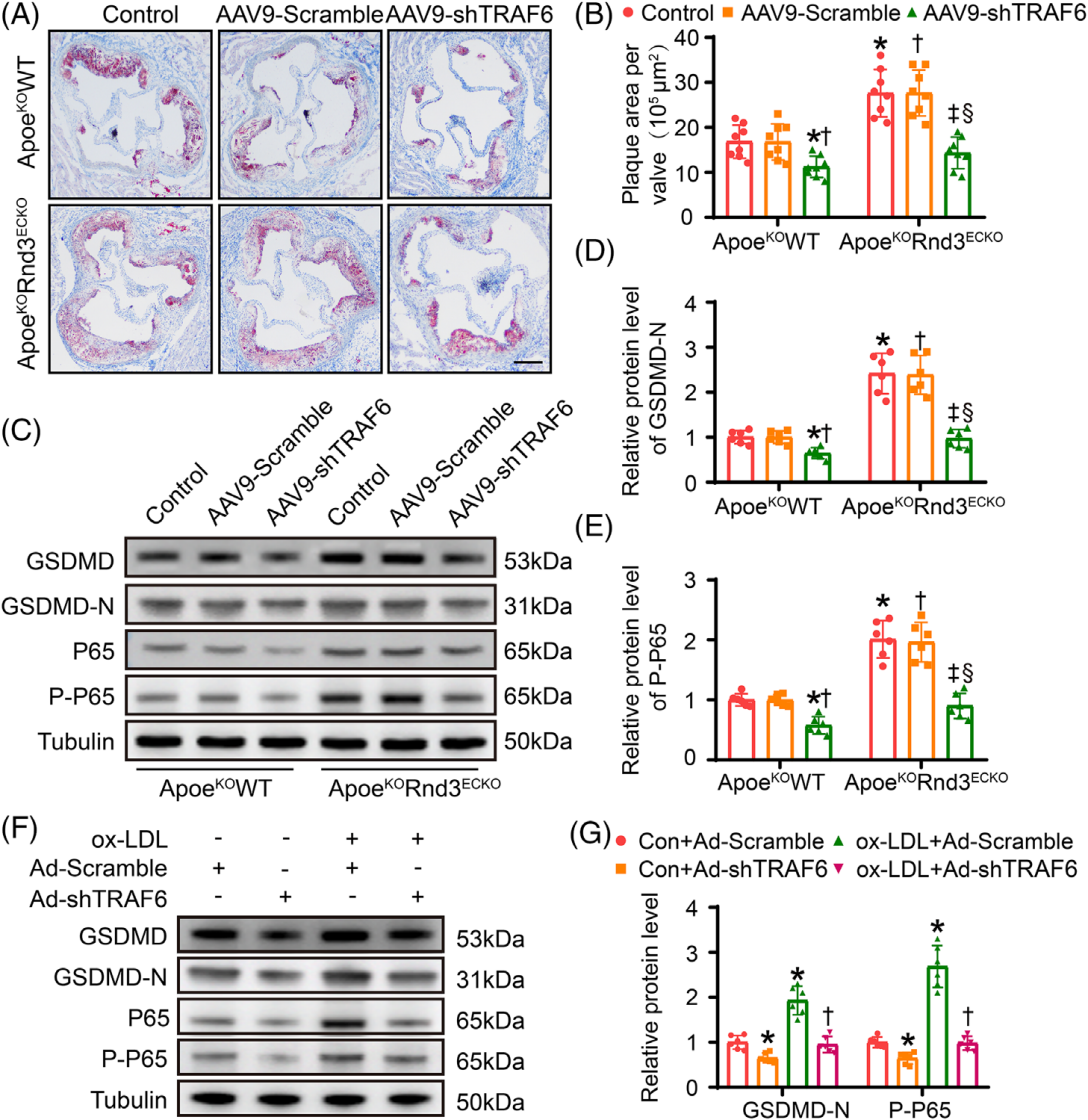
TRAF6 knockdown offsets endothelial pyroptosis and atherosclerosis exacerbated by Rnd3 knockout. (A) Representative Oil Red O staining of aortic roots in different groups (*n* = 10). Scale bars represent 500 μm. (B) Quantification of the percentage of aortic lesion areas in different groups. (C–E) Western blot and quantitative analysis of GSDMD‐N and P‐P65 expression in endothelial cells (ECs) treated as indicated (*n* = 6). *p < .05 versus control in Apoe^KO^WT; **^†^** *p* < .05 versus AAV9‐Scramble in Apoe^KO^WT; **^‡^** *p* < .05 versus control in Apoe^KO^WT; **^§^** *p* < .05 versus AAV9‐Scramble in Apoe^KO^WT; (F and G) western blot and quantitative analysis of gasdermin‐D (GSDMD) and NF‐κB expression in ECs treated as indicated. *p < .05 versus Con+Ad‐Scramble; **^†^** *p* < .05 versus ox‐LDL+Ad‐Scramble.

As expected, Oil Red O staining showed that TRAF6 knockdown mitigated 52.6 ± 3.8% of plaque area formation in Apoe^KO^Rnd3^ECKO^ mice (Figure 6A,B), suggesting a signal interaction between Rnd3 and TRAF6 in ECs during atherosclerosis. Notably, AAV9‐shTRAF6 also reduced plaque formation by 31.8 ± 2.9% in Apoe^KO^WT mice. It was indicated that endothelial cell knockdown of TRAF6 may suppress atherosclerosis itself, in addition to counteracting atherosclerosis aggravated by Rnd3 knockdown. Consistent with these changes, TRAF6 knockdown cancelled out the expression levels of GSDMD‐N and P‐P65 aggravated by Rnd3 knockout (Figure 6C–E). For in vitro experiments, Ad‐shTRAF6 balanced the expression levels of GSDMD‐N and P‐P65 raised by Rnd3 knockout in the face of oxLDL challenge (Figure 6F,G). It is worth noting that the protein levels of Rnd3 remained unchanged regardless of whether TRAF6 was overexpressed or knocked down under oxLDL challenge, so it can be inferred that Rnd3 is not a substrate of TRAF6 ubiquitination (Figure S4I,J). Taken together, the aforementioned in vivo and vitro data further explicitly showed that Rnd3 regulates pyroptosis of ECs during atherosclerosis through TRAF6.

## 4 DISCUSSION

Atherosclerotic disease is a chronic process contributing to acute clinical events, in particularly acute coronary syndrome, myocardial infarction, and stroke. EC injury represents the initial event of atherosclerosis. The salient findings from our study demonstrate an essential role for Rnd3 in ECs during atherosclerotic process. Our results noted downregulated Rnd3 expression with increased endothelial pyroptosis during atherosclerosis progression. Next, we reported a protective effect of EC Rnd3 in atherosclerosis using two animal models. Moreover, Rnd3 deficiency significantly promoted endothelial pyroptosis and plaque formation in Apoe^KO^ mice. Mechanistically, Rnd3 negatively regulated pyroptosis signaling through direct interaction with the RF domain of TRAF6 which inhibited K63‐linked TRAF6 ubiquitination and promoted K48‐linked TRAF6 ubiquitination. These findings reveal the regulatory function of Rnd3 within the TRAF6/NF‐κB/NLRP3 signaling cascade in ECs, highlighting the potential therapeutic value of targeting Rnd3 to combat atherosclerosis.

Pyroptosis of ECs driven by inflammasome NLRP3 is the key pathological process of atherosclerosis.^7^ NLRP3 initiation involves recognition of PAMPs and DAMPs by TLR and activation of NF‐κB.^25^ TLR4/NF‐κB signaling is essential for the transmission of inflammatory signals, while oxLDL can be directly recognized by TLR4 and activate NF‐κB.^26^ Activated NF‐κB has been shown to promote pyroptosis through ROS, endoplasmic reticulum stress, mitochondrial dysfunction, and other mechanisms.^27^ During the activation of NF‐κB, the E3 ubiquitin ligase TRAF6 forms K63‐linked polyubiquitin chains to activate the IKK complex, leading to the phosphorylation of the inhibitor of NF‐κB (IκB) and subsequent degradation.^23^ TRAF6 catalyzes k63‐linked ubiquitination dependent on the RF domain.^17^ The TRAF6 domain's C70 residue has been found to play a crucial role in its ubiquitin ligase activity, and the E3 ubiquitin ligase activity of TRAF6 is compromised by the C70A mutation.^28^ Interestingly, our study showed that Rnd3 interacts with TRAF6 in the 65 to 88 amino acid domains of the RF. The action of Rnd3 may trigger changes in the structure of the C70 site. The K48‐linked ubiquitination usually targets proteins for their subsequent degradation in proteasomes.^23^ In our hands, Rnd3 deficiency reduced TRAF6 degradation, increased TRAF6 protein levels, and exacerbated endothelial pyroptosis and atherosclerosis by reducing K48‐linked ubiquitination of TRAF6. AAV9‐shTRAF6 substantially offset the effect of Rnd3 deficiency, reducing 52.6% of plaque area formation in Apoe^KO^Rnd3^ECKO^ mice. TRAF6 knockdown partially offset endothelial pyroptosis and plaque formation exacerbated by Rnd3 deletion in Apoe^KO^Rnd3^ECKO^ mice, suggesting that Rnd3 may regulate pyroptosis through other undefined mechanisms. Rock1 is the downstream effector of RhoA and can be antagonized by Rnd3.^29^ It has been reported to increase NF‐κB activation through TLR4‐mediated signaling and promote lipopolysaccharide‐induced inflammation of corneal epithelial cells.^30^ Dai et al. found that cardiac Rnd3 overexpression inhibited NF‐κB activity, alleviated post‐myocardial infarction inflammation, as well as improved cardiac function and survival.^11^ Of note, Rnd3 seems to exhibit profound interaction in ECs directly through Rock1 or NF‐κB in atherosclerosis.

It has been reported TRAF6 acts as a bifurcation point of death and survival signals in ECs in the face of lipopolysaccharide challenge.^31^ Lv et al. showed that YAP regulates endothelial activation and vascular inflammation by modulating TRAF6 ubiquitination.^32^ The crucial role of TRAF6 in modulating vascular inflammation in ECs has been supported by several independent studies. Notably, early work on endothelial TRAF6 mainly focused on lipopolysaccharide and high glucose challenge.^33^, ^34^ Our study showed that AAV9‐shTRAF6 reduced plaque formation by 31.8 ± 2.9% in Apoe^KO^ mice, demonstrating a protective role for TRAF6 deficiency on Apoe^KO^ mice. Earlier evidence has denoted that leukocyte deficiency of TRAF6 effectively improved atherosclerosis and restenosis.^35^ These results strongly suggest promises of global TRAF6 inhibition in the treatment of atherosclerosis.

In earlier studies, endogenous antagonism to actin cytoskeleton dynamics mediated by RhoA signaling was deemed to elicit a basic role for Rnd3.^36^ Recent research indicates that Rnd3 can rebalance the signaling of RhoA and Rac1 in ECs, suggesting that delivering Rnd3 may have anti‐inflammatory properties and promote the restoration of endothelial barriers under proinflammatory conditions.^16^ Rnd3 was also identified as a hub gene for peripheral venous congestion causing EC injury (GSE38783 in NCBI GEO).^15^ Here, we noted a rather critical role of Rnd3 as a checkpoint modulator of endothelial injury. Our findings provide compelling evidence that downregulation of Rnd3 sensitizes the ECs to oxLDL stimulation, while elevated Rnd3 levels serve as a protective machinery to restrain inflammation signaling and protect against pyroptosis.

*Experimental limitations*: Several limitations prevail in our study. While Apoe^KO^ and LDL‐receptor (LDLr) knockout models are commonly employed as atherosclerosis mouse models, Apoe3‐Leiden and PCSK9‐AAV models are valuable tools in atherosclerosis research^37^; the critical role of Rnd3 in ECs during atherosclerosis needs to be verified in human and other murine models. Although our results suggest that TRAF6 knockdown substantially counteracts the effect of Rnd3 deficiency, it mainly counteracts the elevated levels of TRAF6 protein evoked by decreased k48 ubiquitination; TRAF6 knockdown is difficult to generate a quantitative relationship with increased k63 ubiquitination. Additionally, it is important to consider that other proteins co‐immunoprecipitated with Rnd3 may have significant involvement in signaling related to inflammation. These proteins should not be overlooked.

In conclusion, findings from our current study have provided the first evidence that Rnd3 regulates pyroptosis of ECs and identifies a new mechanism by which Rnd3 regulates inflammatory signaling, thus providing therapeutic potential for targeting Rnd3 in the treatment of atherosclerosis.

## CONFLICT OF INTEREST STATEMENT

The authors declare no conflicts of interest.

## Supporting information

Supporting Information

## Data Availability

The datasets in this study are available from the corresponding author upon reasonable request.
