# Supplementary material for: Rnd3 suppresses endothelial cell pyroptosis in atherosclerosis through regulation of ubiquitination of TRAF6
Source: Clin Transl Med. 2023 Sep 24;13(9):e1406. doi: 10.1002/ctm2.1406 (PMC10518494; doi:10.1002/ctm2.1406)
Supplement: Supplementary file 1 — Supporting Information [file CTM2-13-e1406-s001.docx]

**Supplemental Data**

**Experimental Animals**

In brief, endothelial-specific knockout mice (Rnd3ECKO) were generated by crossing Rnd3 flox homozygous mice (fl/fl) with Cdh5-CreERT2 mice. Endothelial-specific transgenic mice (Rnd3ECTG) were generated by crossing conditional Rosa26 Rnd3 knockin homozygous (lsp/lsp) mice with Cdh5-creERT2 mice. Then mice were crossed with Apolipoprotein E knockout (ApoeKO) mice to yield Rnd3ECKO-ApoeKO and Rnd3ECTG-ApoeKO mice. PCR was employed to determine the genotype of mice. Following genotyping, mice were administrated tamoxifen (75mg/kg) for five consecutive days. The mice were housed in our animal facility under a 12-hour light/12-hour dark circadian cycle with free access to food and water, while maintaining a temperature of 22°C ± 1°C and a humidity level of 60 ± 5%. All mice were fed a high-fat diet containing 40% of kcal from fat, 43% of kcal from carbohydrates, and 17% of kcal from protein, manufactured by Beijing Hfk Bioscience Co. Ltd., Beijing, China, starting at 8 weeks of age and continuing for a duration of 8 weeks. Rnd3fl/flKO mice were purchased from Shanghai Model Organisms Center, Inc. (serial number: 2018-W-3735, China). Rnd3lsp/lspTg mice were acquired from Cyagen Biosciences Inc. (serial number: TOS161121BA2, China). Cdh5-creERT2 and ApoeKO mice were obtained from Gempharmatech Co., Ltd. (serial number: GJS1910160298; GJS2005270118, China).

**Gene intervention**

ECs were cultured with serum-free medium 24 hours for synchronization when cells reached 50-60% confluence. The adenoviruses harboring Rnd3 (Ad-Rnd3, Hanbio Technology, Shanghai, China), shRnd3 (Ad-shRnd3, Hanbio Technology), shTRAF6 (Ad-shTRAF6, Tsingke Biotechnology, Shanghai, China) and control vectors with different multiplicities of infection (MOIs) were transfected into ECs for 8 h. Following 8 h of infection, medium was replaced with a fresh medium containing 10% FBS for another 36 h. Western blotting was employed to evaluate transfection efficiency of adenoviruses.

To establish an endothelial cell (EC)-specific TRAF6 knockdown mouse model, mouse TRAF6 knockdown adeno-associated virus-9 genome particles containing the TIE promoter, Flag and EGFP (AAV9-m-TIE-shTRAF6-Flag-EGFP, abbreviated to AAV9-shTRAF6) were conducted by Tsingke Biotechnology. 100 µl AAV9-shTRAF6 or AAV9-Scramble at a density of 1.8 × 10^12 v.g./ml was delivered into 8-week-old C57BL/6J mice through the caudal vein. Four weeks after transfection, cardiac tissues were sectioned to measure transfection efficiency of AAV9 at the root of the aorta via co-immunofluorescence staining of Flag and CD31.

The sequences of shRNA for Rnd3 is 5’-CCAGAGACTCTGGACAGTGTCTTAATTCAAGACACTGTCCAGAGTCTCTGGTT-3’. The sequences of shRNA for TRAF6 is 5’-CCGGCCCAGGCTGTTCATAATGTTACTCGAGTAACATTATGAACAGCTGGGTTTTTT-3’.

**Immunofluorescence staining**

Frozen sections were permeabilized with 0.5% Triton X-100, and were blocked with 1% bovine serum albumin (BSA) at 37°C for 1 hour. Then sectioned samples were incubated with the primary antibody at 4°C for 16 hours, and were rinsed three times with PBS for 10 minutes each. Samples and secondary antibodies were given another hour at 37 °C. After washing with PBS, samples were incubated in DAPI working solution at 37°C for 10 minutes. Finally, antifade solution were added and the acquired images were visualized under a fluorescence microscope (Nikon Eclipse C1, Tokyo, Japan). (Refer to the supplementary materials for the antibodies used and the corresponding dilution ratios).

**Western blot**

Following isolation of aortic endothelial cells from mice as described above, cells were resuspended with PBS and were centrifuged at 1000 RPM for 5 min, repeated three times. The RIPA containing protease inhibitor and phosphatase inhibitor were added to cell pellet at 4 °C for 30 min. Protein concentration was determined using BCA method. Cells cultured in six-well plates were collected by cell spatula. Protein was separated by SDS-PAGE, and transferred to nitrocellulose membranes. After blocking for 1 hour at 37°C with 5% skimmed milk, the blots were incubated with the respective primary antibodies for 8 –12 h at 4°C and with secondary HRP-conjugated antibodies for 1 h at 37°C. Finally, the antigen-antibody complexes were detected by a chemiluminescence system (Amersham Bioscience, Buckinghamshire, UK) as previously described39. The antibody information and the details are shown in supplementary table 1 (sTable 1).

**qPCR**

Total RNA of ECs from mice or cultured six-well plates was extracted using TRIzol (Invitrogen), and reverse transcribed into cDNA using the PrimeScriptRT Reagent Kit (TaKaRa, Dalian, China). qPCR was performed using SYBR Green (Bio-Rad Laboratories, CA, USA). All procedures were performed in accordance with the manufacturer's instructions. The primer sequences for qPCR were listed in sTable. 2.

sTable 1. Primary antibodies used for western blotting, immunoprecipitation, **and** immunohistochemistry

| Antibody | Working dilutions | Catalog No. | Supplier |
| --- | --- | --- | --- |
| Rnd3 | IF: 1/200 | 05-723 | Sigma-Aldrich, MO, USA |
| Rnd3 | WB: 1/1000 | ab171799 | Abcam, Cambridge, UK |
| GSDMD | WB: 1/1000  IF: 1/200 | PA5-116815 | Invitrogen, CA, USA |
| CD31 | IF: 1/200 | 14-0311-82 | Invitrogen, CA, USA |
| GSDMD-N | WB: 1/1000 | 10137 | CST, MA, USA |
| NLRP3 | WB: 1/1000 | #4147 | Adipogen, CA, USA |
| Pro-caspase1 | WB: 1/1000 | ab179515 | Abcam, Cambridge, UK |
| Caspase1 | WB: 1/1000 | # 89332S | CST, MA, USA |
| Tubulin | WB: 1/5000 | AC008 | ABclonal, Wuhan, China |
| TRAF6 | WB: 1/1000  IP:1/200 | ab137452 | Abcam, Cambridge, UK |
| Ubiquitin | WB: 1/1000 | ab134953 | Abcam, Cambridge, UK |
| Ub-K48 | WB: 1/1000 | ab140601 | Abcam, Cambridge, UK |
| Ub-K63 | WB: 1/1000 | ab179434 | Abcam, Cambridge, UK |
| P-P65 | WB: 1/1000 | ab76302 | Abcam, Cambridge, UK |
| P65 | WB: 1/1000 | ab16502 | Abcam, Cambridge, UK |
| Myc | WB: 1/1000  IP:1/200 | #2276 | CST, MA, USA |
| Flag | WB: 1/1000  IP:1/50  IF:1/200 | #8146 | CST, MA, USA |
| Ha | WB: 1/1000 | #3724 | CST, MA, USA |

sTable 2. Mass spectrum analysis of top 10 Rnd3-interacting proteins

| Protein name | peptide spectrum matches | Unique peptides | MW [kDa] | Abundances |
| --- | --- | --- | --- | --- |
| Traf6 | 21 | 18 | 60.2 | 1.4 × 10^8^ |
| Trim29 | 15 | 13 | 65.8 | 5.3 × 10^7^ |
| Irf2bp1 | 10 | 8 | 61.7 | 2.1 × 10^7^ |
| Gja10 | 9 | 8 | 57.1 | 1.9 × 10^7^ |
| Atp5f1a | 6 | 5 | 59.7 | 6.5 × 10^6^ |
| Cyp2u1 | 6 | 4 | 60.5 | 4.1 × 10^6^ |
| Tent4b | 5 | 3 | 69.7 | 2.8 × 10^6^ |
| Ncoa5 | 3 | 2 | 65.3 | 7.5 × 10^5^ |
| Dmrt2 | 3 | 1 | 61.6 | 3.6 × 10^5^ |
| Sf3a3 | 2 | 1 | 58.8 | 2.3 × 10^5^ |

sTable 3. Primer sequences for RT-PCR

| Mouse | Forward 5’ to 3’ | Reverse 5’ to 3’ |
| --- | --- | --- |
| Rnd3 | TTTCGCACATGCCTAGCAGA | CAGATATTCCCGCGTCCTCC |
| NLRP3 | AGACTGACGTCTCCGCTTTC | CTGGTCCTTTCCTCACGGT |
| Pro-caspase1 | CGTACACGTCTTGCCCTCAT | GGGCAGGCAGCAAATTCTTT |
| GSDMD | AGTGCTCCAGAACCAGAACCG | TCTCCCATGCCTGACAACATC |
| GAPDH | GGTGAAGGTCGGTGTGAACG | CTCGCTCCTGGAAGATGGTG |


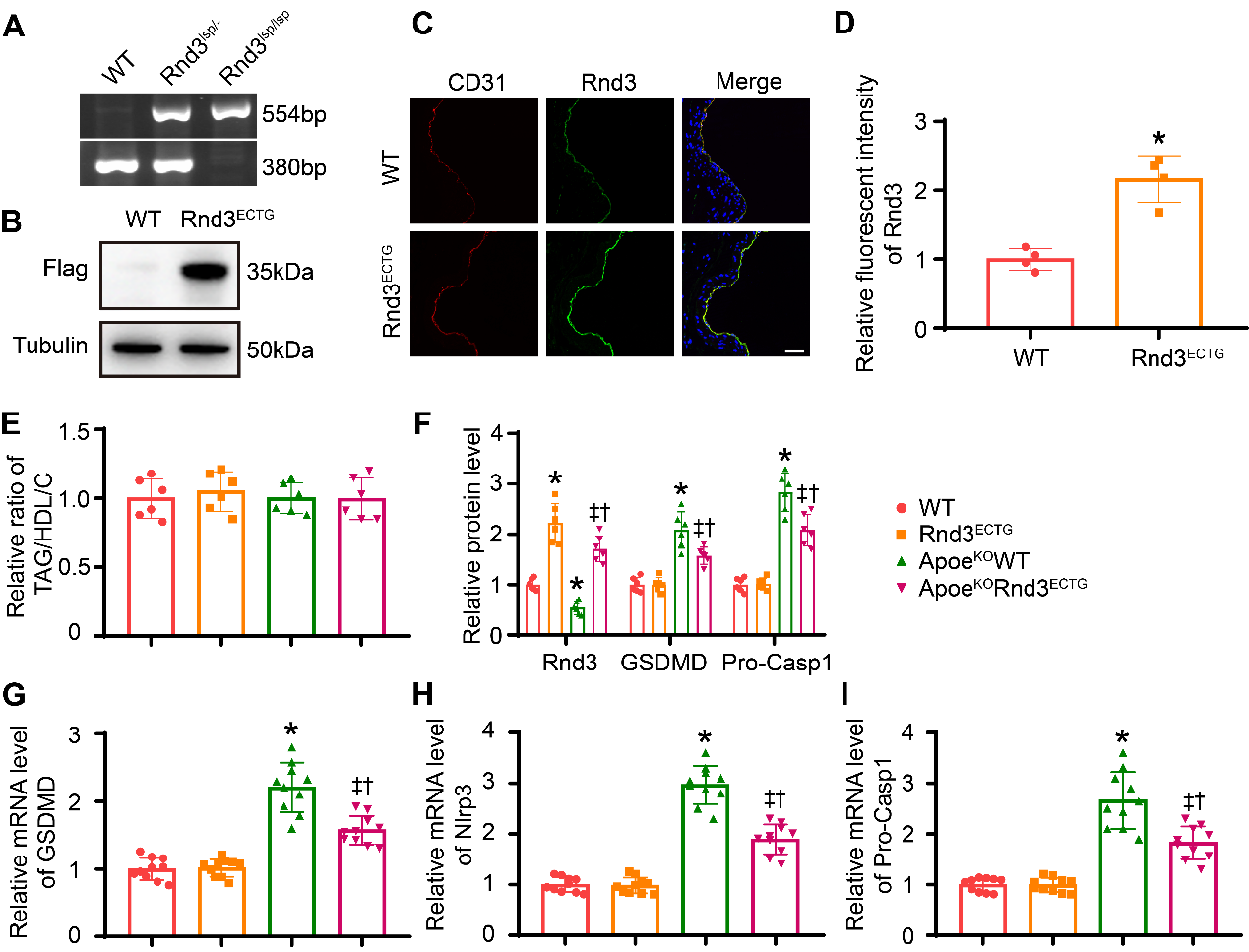


**sFigure 1. Overexpression of Rnd3 inhibits endothelial pyroptosis in Apoe^KO^ mice.** (A)

Genotype of mice was identified by PCR. (B) Western blot was employed to confirm the specificity of Rnd3 overexpression in ECs. (C-D) Immunofluorescence was employed to evaluate the protein levels of Rnd3 in ECs (n = 4). Scale bars represent 50 μm. **P* < 0.05 *vs.* WT; (E) The ratio of triglyceride to high-density lipoprotein cholesterol (TAG/HDL-C) in each group of mice (n = 6). (F) Relative protein level of Rnd3, GSDMD, and Pro-Caspase1 in each group of mice (n = 6). (G-I) qPCR analysis of NLRP3, GSDMD, and Pro-Caspase1 in different groups (n = 10). **P* < 0.05 *vs.* WT; **^†^***P* < 0.05 *vs.* Rnd3^ECTG^; **^‡^***P* < 0.05 *vs.* ApoE^KO^WT.


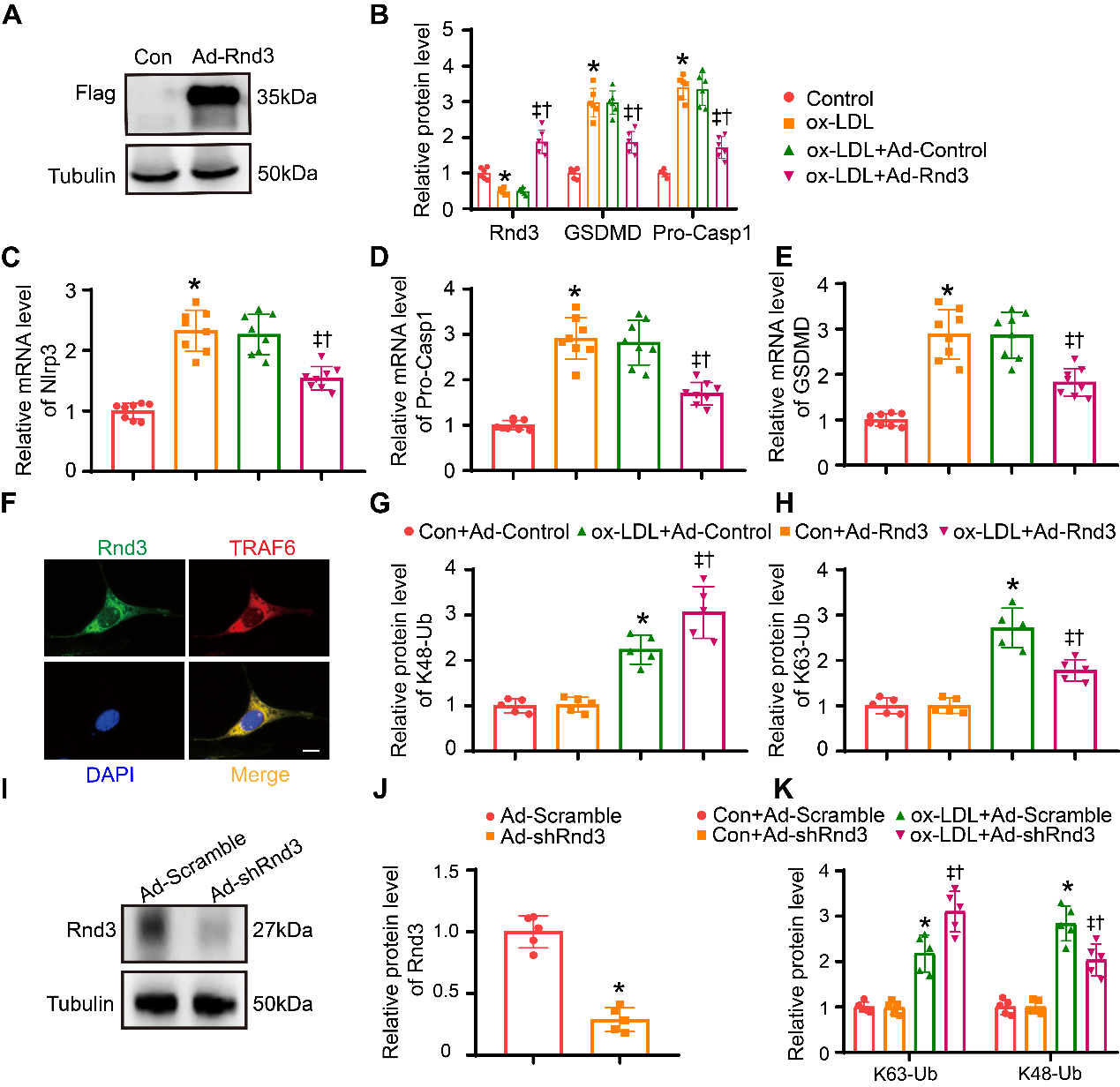


**sFigure 2. Rnd3 inhibits endothelial pyroptosis by regulating the ubiquitination of TRAF6.** (A) Western blot was employed to confirm the specificity of Rnd3 overexpression in ECs. (B) Relative protein levels of Rnd3, GSDMD, and Pro-Caspase1 in each group (n = 6). (C-E) qPCR analysis of NLRP3, GSDMD, and Pro-Caspase1 in different groups (n = 10). **P* < 0.05 *vs.* Control; **^†^***P* < 0.05 *vs.* oxLDL; **^‡^***P* < 0.05 *vs.* oxLDL+Ad-Control. (F) Immunofluorescence staining of Rnd3 (green), TRAF6 (red) in mouse primary aortic ECs. Scale bars represent 20 μm. (G-H) The protein level of K63-Ub, and K48-Ub after TRAF6 IP was evaluated by western blot (n=5). **P* < 0.05 *vs.* Con+Ad-Control; **^†^***P* < 0.05 *vs.* Con+Ad-Rnd3; **^‡^***P* < 0.05 *vs.* oxLDL+Ad-Control. (I) Western blot was used to evaluate the protein level of Rnd3 in ECs as treated. (J) Relative protein levels of Rnd3 in each group (n = 5). **P* < 0.05 *vs.* Ad-Scramble; (K) The protein level of K63-Ub, and K48-Ub after TRAF6 IP in each group was evaluated by western blot (n=5). **P* < 0.05 *vs.* Con+Ad- Scramble; **^†^***P* < 0.05 *vs.* Con+Ad-shRnd3; **^‡^***P* < 0.05 *vs.* oxLDL+Ad-Scramble.


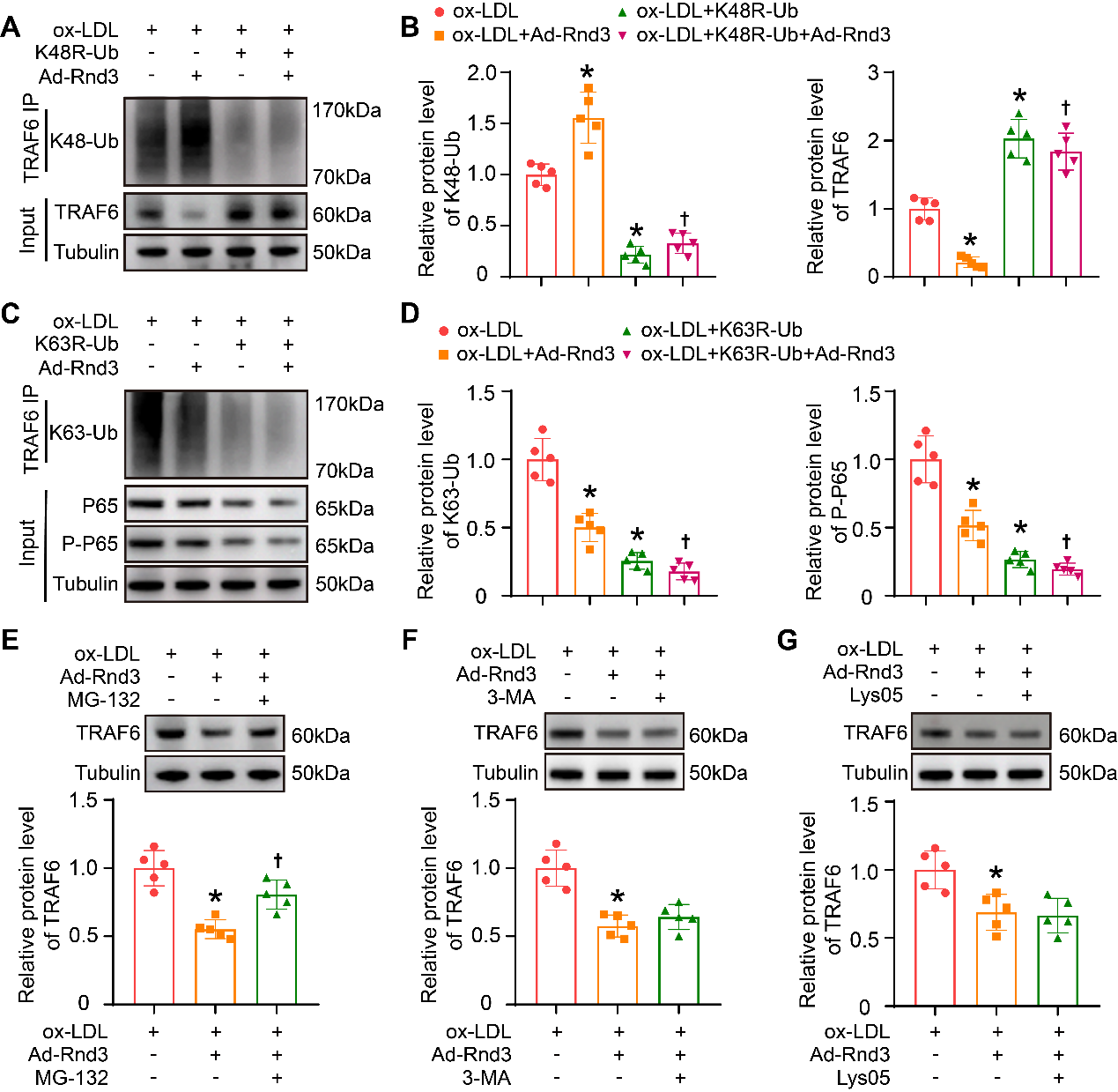


**sFigure 3. Rnd3 inhibits endothelial pyroptosis by regulating the ubiquitination of TRAF6.** (A-B) Western blot and quantitative analysis of K48-Ub and TRAF6 expression in ECs treated as indicated (n=5). **P* < 0.05 *vs.* ox-LDL; **^†^***P* < 0.05 *vs.* ox-LDL+Ad-Rnd3. (C-D) Western blot and quantitative analysis of K63-Ub and P-P65 expression in ECs treated as indicated (n=5). **P* < 0.05 *vs.* ox-LDL; **^†^***P* < 0.05 *vs.* ox-LDL+Ad-Rnd3. (E-G) The TRAF6 protein level was assessed via western blot analysis according to the indicated treatments (n=5). **P* < 0.05 *vs.* ox-LDL; **^†^***P* < 0.05 *vs.* ox-LDL+Ad-Rnd3.


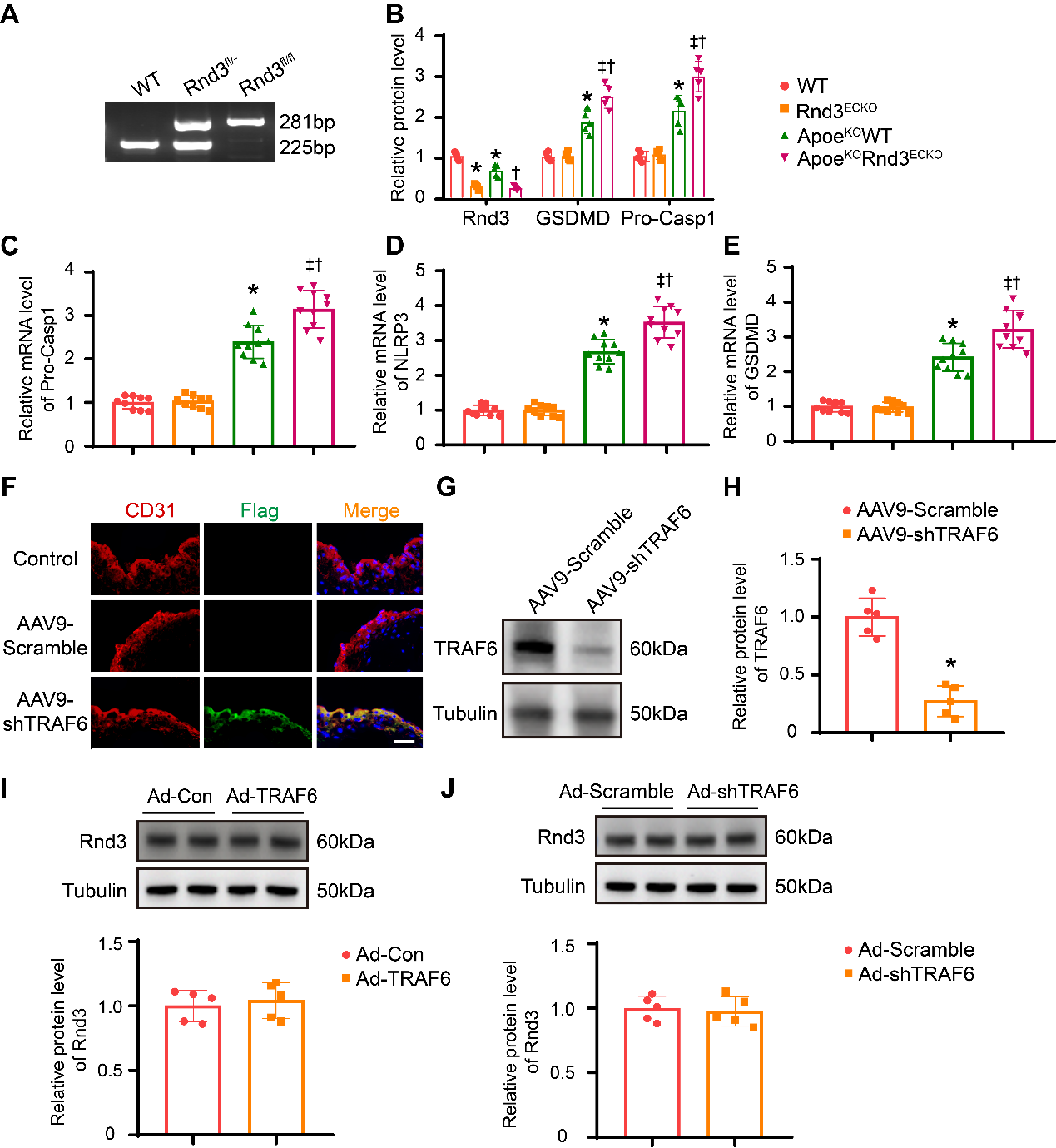


**sFigure 4. TRAF6 knockdown offsets endothelial pyroptosis and atherosclerosis exacerbated by Rnd3 knockout.** (A) Genotype of mice was identified by PCR. (B) Relative protein levels of Rnd3, GSDMD, and Pro-Caspase1 in each group (n = 5). (C-E) qPCR analysis of NLRP3, GSDMD, and Pro-Caspase1 in different groups (n = 10). **P* < 0.05 *vs.* WT; **^†^***P* < 0.05 *vs.* Rnd3^ECKO^; **^‡^***P* < 0.05 *vs.* ApoE^KO^WT. (F) Immunofluorescence staining of Flag and CD31 in aortic root was used to evaluate the transfection efficiency of AAV9. Scale bars represent 100 μm. (G-H) Western blot and quantitative analysis of TRAF6 expression in ECs treated as indicated. **P* < 0.05 *vs.* AAV9-Scramble. (I) Western blot and quantitative analysis of Rnd3 expression in ECs treated as indicated (n=5). **P* < 0.05 *vs.* Ad-Con; (J) Western blot and quantitative analysis of Rnd3 expression in ECs treated as indicated (n=5). **P* < 0.05 *vs.* Ad-Scramble;
